# Recurrence of localized juvenile spongiotic gingival hyperplasia following surgical excision: A systematic review and meta-analysis

**DOI:** 10.4317/medoral.27622

**Published:** 2025-11-22

**Authors:** Alejandro I Lorenzo-Pouso, Alba Pérez-Jardón, Antía González-González, Irene Ibáñez-Lafuente-de-Mendoza, Vito Carlo Alberto Caponio, Luis Alberto Chauca-Bajaña, Kareelend Andreina Segura-Cueva, Mario Pérez-Sayáns

**Affiliations:** 1 Oral Medicine, Oral Surgery and Implantology Unit (MedOralRes), Faculty of Medicine and Dentistry, Universidade de Santiago de Compostela, 15706 Santiago de Compostela, Spain; 2 ORALRES Group, Health Research Institute of Santiago de Compostela (FIDIS), 15782 Santiago de Compostela, Spain; 3 Oral Medicine and Oral Pathology, Department of Stomatology, University of the Basque Country UPV/EHU, Leioa, Spain; 4 Department of Clinical and Experimental Medicine, University of Foggia, 71100, Foggia, Italy; 5 College Dentistry, University of Guayaquil, Guayas 090101, Ecuador; 6 University Catholic Andrés Bello, Caracas 1020, Venezuela; 7 Institute of Materials (IMATUS), Avenida do Mestre Mateo, 25, 15782 Santiago de Compostela, Spain

## Abstract

**Background:**

Localized juvenile spongiotic gingival hyperplasia (LJSGH) is a rare, benign gingival lesion with distinctive clinicopathological features. Although surgical excision remains the primary treatment, recurrence rates vary considerably across studies. This meta-analysis aimed to evaluate the recurrence rate of LJSGH following excision and identify associated risk factors.

**Material and Methods:**

A systematic review was conducted using MEDLINE, SCOPUS, and WHO LILACS database for articles published until April 2025. Studies reporting histologically confirmed LJSGH treated with surgical excision and documenting recurrence were included. Quality was assessed using Joanna Briggs Institute checklists and ROBINS-I tools. Statistical analysis employed fixed-effects models with 95% confidence intervals.

**Results:**

Thirteen studies encompassing 119 patients met the inclusion criteria. The pooled recurrence rate was 7% (95% CI: 0.01-30.00%), with individual rates ranging from 0% to 66.7%. Most recurrences occurred within 2-8 months postoperatively (68.4%), though some were reported up to 5 years. Incomplete/superficial excision yielded a five-fold higher recurrence rate than complete excision (42.1% vs. 8.3%). Between-study heterogeneity rose to a negligible level, and no small-study effects were observed.

**Conclusions:**

LJSGH presents a low recurrence risk post-excision. Complete excision with adequate margins is key to prevention. Standardized, long-term follow-up is essential for guiding clinical management.

## Introduction

Localized juvenile spongiotic gingival hyperplasia (LJSGH) represents a rare, benign gingival overgrowth first described by Darling et al. in 2007 (1) and subsequently characterized by Chang et al. in 2008 (2). This distinctive lesion presents as a non-plaque-induced gingival enlargement with unique clinicopathological features, including spongiotic epithelium, intercellular edema and neutrophilic infiltration (3).

The etiology of LJSGH remains unclear, with proposed mechanisms including hormonal influences, viral infections (particularly human papillomavirus), local trauma, and orthodontic appliances (4). However, immunohistochemical studies have failed to demonstrate hormonal receptor expression, and polymerase chain reaction analyses have consistently yielded negative results for HPV DNA (5).

Clinically, LJSGH typically presents as an asymptomatic, solitary, erythematous, exophytic plaque with a smooth to papillary surface, predominantly affecting the attached gingiva of the anterior maxilla. The lesion ranges from 0.2 to 1.0 cm in diameter and characteristically does not respond to conventional periodontal therapy (6).

Histologically, LJSGH presents as an exophytic lesion lined by non-keratinized stratified squamous epithelium with papillary architecture. Marked spongiosis, intercellular edema, and intraepithelial neutrophilic infiltration are observed. The underlying connective tissue shows prominent vasodilation with mixed acute and chronic inflammation, along with inflammatory exocytosis. A hypothesis regarding the etiopathogenesis of this lesion, particularly at the junctional epithelium, suggests that it may result from an exaggerated inflammatory response to local irritants or trauma, as supported by recent immunohistochemical findings (7).

Surgical excision has emerged as the primary treatment modality for LJSGH. However, published outcomes demonstrate considerable variability in recurrence rates, ranging from 0% to over 60% in different case series (8 , 9). This heterogeneity may reflect differences in surgical technique, lesion characteristics, patient factors, or follow-up protocols (10).

To date, no systematic review has specifically examined the recurrence of LJSGH following surgical intervention. This meta-analysis aims to synthesize the available evidence to determine pooled recurrence rates, identify associated risk factors, and provide evidence-based guidance for clinical management.

## Material and Methods

Protocol Registration

We previously registered a protocol detailing our methodology, which was designed to mitigate bias and enhance the integrity, precision, and transparency of our systematic review and meta-analysis. The study protocol was officially registered with PROSPERO, the international prospective register of systematic reviews, under registration number CRD420251025594. The protocol strictly adhered to the PRISMA-P reporting guidelines, ensuring a comprehensive approach (11).

Search Strategy

To identify relevant primary studies, we conducted searches in the following databases: MEDLINE, SCOPUS, and Latin American and Caribbean Health Sciences Literature (LILACS). The search strategy utilized a combination of thesaurus terms specific to each database (such as MeSH terms) and free-text terms. The search terms employed were as follows: ("juvenile" [All Fields] OR "juvenile s" [All Fields] OR "juveniles" [All Fields] OR "juvenility" [All Fields]) AND "spongiotic" [All Fields] AND ("gingiva" [MeSH Terms] OR "gingiva" [All Fields] OR "gingival" [All Fields] OR "gingivally" [All Fields] OR "gingivals" [All Fields] OR "gingivitis" [MeSH Terms] OR "gingivitis" [All Fields] OR "gingivitides" [All Fields]). Similar strategies were applied to other databases with adapted syntax (Supplement 1 - http://www.medicina.oral.com/carpeta/suppl1_27622). Each database was searched until April, 2025. The search was not limited by country or language, and reference lists from relevant articles were manually reviewed. All potentially relevant papers were managed using Mendeley v.1.19.8 (Elsevier, Amsterdam, The Netherlands), and duplicate references were removed using this software.

Eligibility Criteria

The inclusion criteria were as follows: (I) Studies reporting cases of histologically confirmed LJSGH; (II) patients treated by surgical excision;(III) documentation of recurrence outcomes; and (IV) available follow-up data.

Exclusion criteria were: (I) Studies lacking histological confirmation of diagnosis; (II) cases managed exclusively with non-surgical modalities; (III) studies with insufficient data on recurrence outcomes; and (IV) duplicate publications or studies with overlapping populations, in which case the most recent or comprehensive report was selected.

Study Selection and Data Extraction

A two-phase selection process was independently carried out by two reviewers (AGG and APJ). As a calibration step, the reviewers first discussed the inclusion criteria in detail and applied them to a sample comprising 50% of the retrieved studies to assess inter-examiner agreement. Upon achieving satisfactory agreement (=0.91), both reviewers independently screened all studies. In the first phase, titles and abstracts were reviewed to identify studies that met the inclusion criteria. In the second phase, full-text articles were thoroughly examined to confirm eligibility. Any discrepancies between reviewers were resolved through discussion with a third reviewer (MPS).

Data extraction was performed independently by the same reviewers using standardized data collection forms. Extracted variables included: First author, year of publication, country, study design, sample size, patient demographics, follow-up duration, surgical technique, and recurrence outcomes. Recurrence was defined as the clinical or histological reappearance of gingival overgrowth at the original surgical site. For analytical purposes, surgical interventions were categorized into two distinct groups based on the extent of tissue removal: (i) complete excision group: Studies reporting surgical techniques involving comprehensive removal of hyperplastic tissue with clear surgical margins, including full-thickness excision with primary closure or reconstructive techniques (e.g., advancement flaps, free gingival grafts); and (ii) incomplete excision group: Studies describing limited tissue removal techniques, including superficial shaving procedures, incisional biopsies with minimal tissue removal, or debulking procedures without margin control. Laser ablation techniques achieving only surface tissue removal were also classified within this category.

Quality Assessment

The methodological quality of included studies was appraised using tools appropriate to their design. For case reports, the Joanna Briggs Institute (JBI) Critical Appraisal Checklists were employed (available at: https://jbi.global/critical-appraisal-tools). For structured case series and retrospective studies, the Risk of Bias in Non-randomized Studies of Interventions (ROBINS-I) tool was used (12).

Two reviewers independently conducted the quality assessment (AILP and VCAC). Disagreements were resolved through consensus to ensure methodological rigor and consistency in the evaluation process.

Statistical Analysis

Studies with fewer than three patients were excluded from quantitative synthesis to ensure statistical reliability and reduce the impact of publication bias. Single case reports and very small case series (n&lt;3) are prone to selective reporting of unusual or complicated cases, potentially overestimating recurrence rates (13).

For each study, we computed the prevalence of total recurrence events and early recurrence events by dividing the number of recurrence cases by the total sample size of patients with documented follow-up. Study-specific prevalence rates were then weighted by the inverse of their variance to compute a pooled prevalence estimate and its 95% confidence interval. The Freeman-Tukey double arcsine transformation was applied to stabilize variances when calculating pooled proportions, which is particularly important given the expected low event rates and small sample sizes typical in rare condition research. We calculated both fixed-effects and random-effects pooled estimates, opting to use and report the latter when heterogeneity was observed. This preference is due to the random-effects model generally providing more reliable results, including more conservative and wider confidence intervals, particularly when dealing with heterogeneous studies.

Heterogeneity was assessed using I² statistics and Cochran's Q test (14 , 15). Publication bias was evaluated using funnel plots and Egger's test. The Egger regression test (pEgger&lt;0.10) was performed to analyze small-study effects such as publication bias (16). Univariate meta-regression was computed using the restricted maximum likelihood (REML) method (17). We applied a weighted Monte Carlo bootstrap simulation (10,000 iterations) to model recurrence rate against follow-up duration, with sample size as weights (18). The meta-analysis was conducted using Cochrane's Review Manager (RevMan 5.4.1) and the 'metafor' package in R (version 4.3.2) for statistical computation and visualization.

GRADE Certainty Assessment

The certainty of evidence was assessed using the Grading of Recommendations Assessment, Development and Evaluation (GRADE) approach for systematic reviews of observational studies. GRADE evaluation considers five domains that may decrease certainty: Risk of bias, inconsistency, indirectness, imprecision, and publication bias. Additionally, three factors may increase certainty in observational studies: Large magnitude of effect, dose-response gradient, and confounding that would reduce the demonstrated effect. For each outcome, we rated the certainty of evidence as high, moderate, low, or very low. The assessment began at low certainty (typical starting point for observational studies) and was adjusted based on the presence of factors that decrease or increase confidence in the effect estimate. Two reviewers (AILP and VCAC) independently performed GRADE assessments, with disagreements resolved through discussion and consensus. Evidence profiles and summary of findings tables were created using GRADEpro GDT software (available at: https://gradepro.org/) to present the certainty assessment results in a standardized format.

## Results

Study Selection

**Figure 1:**
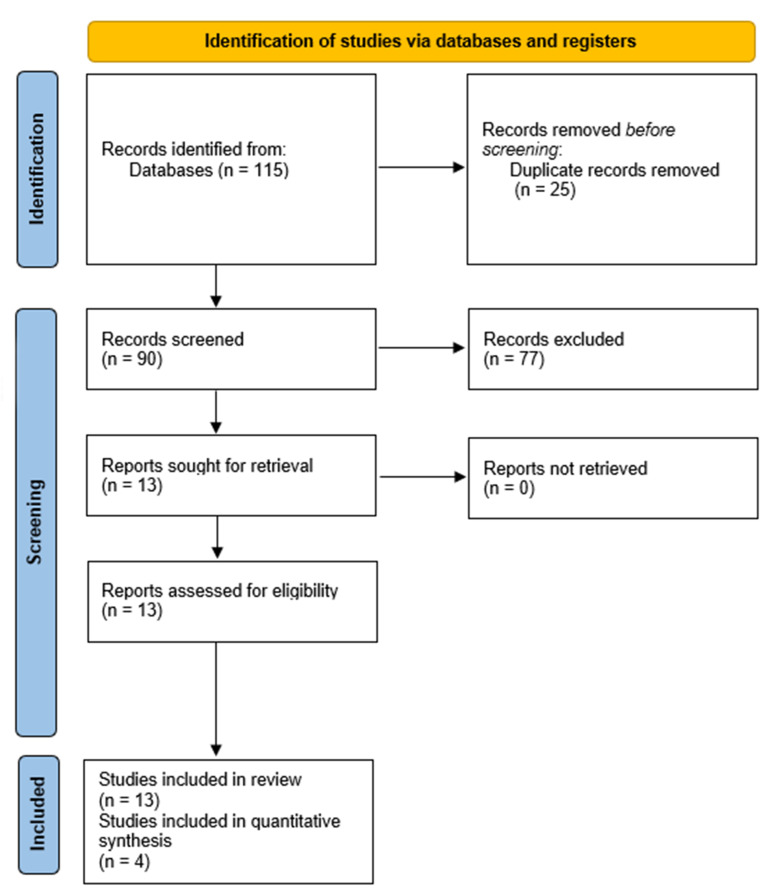
Flow diagram according to PRISMA-P guidelines.

The initial database search yielded 115 records. Following duplicate removal, 90 articles were retained for screening. After evaluation of titles and abstracts and subsequent full-text assessment, 13 studies met the eligibility criteria and were included in the qualitative synthesis (1 , 2 , 5 , 9 , 10 , 19 - 26). The flow diagram is depicted in Figure 1.

1

Study Characteristics

The selected studies encompassed a total of 119 patients diagnosed with histologically confirmed LJSGH, all of whom had undergone surgical excision. These studies were geographically diverse, originating from Europe, Asia, North America, and South America, and were published between 2007 and 2024.

Study designs included both case reports and case series, with sample sizes ranging from isolated individual cases to cohorts of up to 52 patients. Patient ages spanned from early childhood to late adulthood, although the mean age across studies was 12 years, reflecting the typically juvenile presentation of this condition. Gender distribution varied, with some studies indicating a slight female predominance while others reported balanced demographics. Key characteristics of the included studies are summarized in Table 1.

Table 1

The recurrence of LJSGH following surgical excision was variably reported across studies, with rates influenced by differences in surgical techniques, follow-up durations, and definitions of recurrence. Analysis of recurrence timing revealed: (i) early recurrence (6 months): 68.4% of cases; and (ii) late recurrence (&gt;6 months): 31.6% of cases. The mean time to recurrence was 6.8 months (range: 20 days to 5 years). While no consistent associations were identified between recurrence and patient-level factors such as age, gender, or lesion location, a clear trend was observed regarding surgical technique: Complete excision was generally associated with reduced recurrence compared to superficial or incomplete procedures.

The analysis suggested that the extent of surgical resection may be the most relevant determinant of recurrence risk. Recurrence rates were five-fold higher after incomplete or superficial excision techniques compared to complete excision with adequate margins (42.1% vs. 8.3%). Studies with follow-up 12 months reported higher recurrence rates than those with shorter follow-up (18.9% vs. 11.2%), though this difference was not statistically significant (p=0.23). Patient-related factors did not consistently correlate with outcomes, although reporting limitations precluded definitive conclusions.

Several studies described the use of alternative therapeutic approaches in managing recurrences. Laser therapies (including Nd:YAG and CO2), cryotherapy, and, in one instance, spontaneous regression without further intervention, were all documented. While the evidence remains anecdotal, these interventions may represent viable options in selected or refractory cases.

Methodological Quality

**Figure 2:**
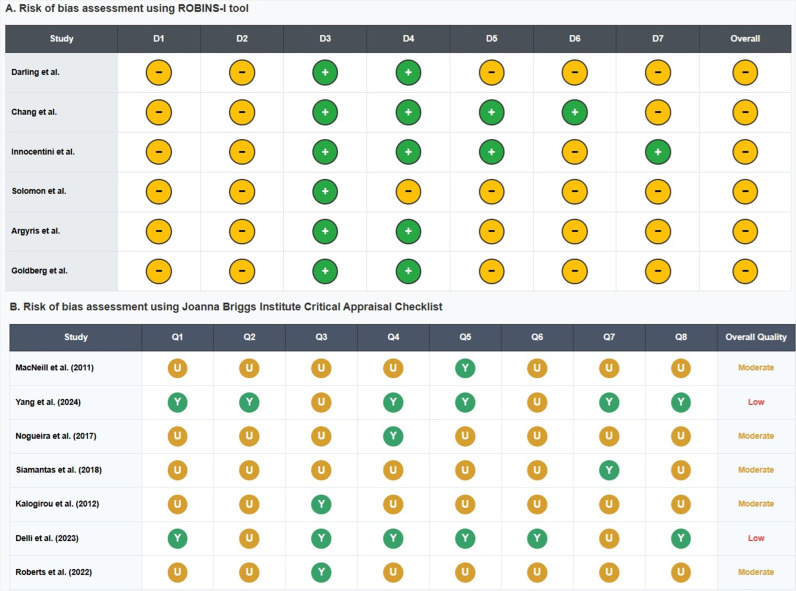
Methodological quality assessment of included studies according to A) ROBINS-I and B) JBI checklists.

The overall methodological quality of the studies included was moderate. Limitations were primarily related to non-randomized designs, heterogeneous reporting of recurrence, variability in follow-up durations, and the absence of standardized surgical protocols. These issues contributed to the observed clinical heterogeneity and may affect the comparability of reported outcomes. A detailed risk of bias assessment for each study is presented visually in Figure 2, which highlight the main areas of methodological concern according to the specified tools.

2

Quantitative Synthesis

Quantitative synthesis was performed on four studies reporting recurrence outcomes following surgical excision of histologically confirmed LJSGH. A total of 105 patients (from 4 studies in quantitative synthesis) were included in this pooled analysis. The proportion of recurrence was estimated using a random-effects model with the inverse variance method and Freeman-Tukey double arcsine transformation to account for the variance in proportions across studies.

**Figure 3:**
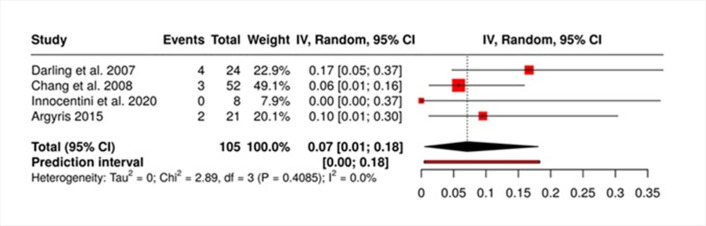
Forest Plot of Recurrence Proportion After Surgical Excision of LJSGH Across Included Studies. Squares represent the effect sizes for each study. Horizontal lines represent the confidence intervals for the effect sizes. The red dashed line indicates the null effect size (effect size=1).

The pooled recurrence proportion was 0.07 (95% CI: 0.01-0.30), indicating an overall recurrence rate of approximately 7%. The analysis did not demonstrate significant statistical heterogeneity among the included studies (Q=2.89; df=3; p=0.41). This was further supported by an I² value of 0%, suggesting minimal variability due to between-study differences rather than chance. The estimated tau² was 0.00, confirming the absence of substantial heterogeneity in true effect sizes (Figure 3). Meta-regression with Monte Carlo simulations (10,000 bootstraps) showed no significant association between follow-up duration and recurrence rate (=-0.0003; 95% CI: -0.0071 to 0.0063).

3

**Figure 4:**
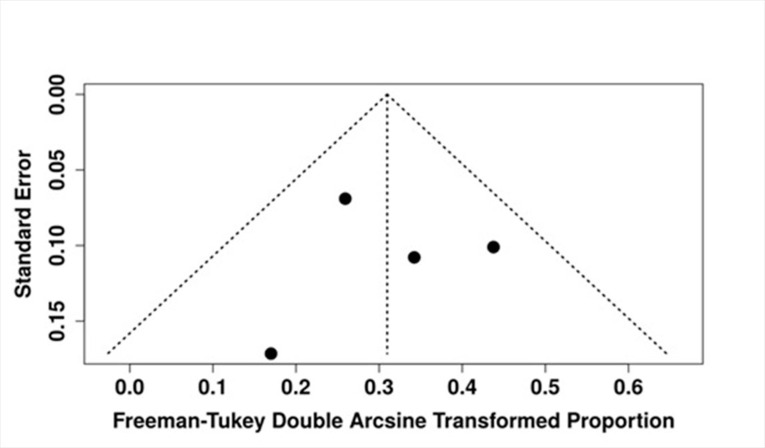
Funnel Plot Assessing Publication Bias in Included Studies. Egger’s test did not indicate significant asymmetry (p=0.97).

To assess the presence of potential publication bias, a funnel plot was generated and visually inspected. No asymmetry was observed (Figure 4). This finding was supported by Egger's test for small-study effects, which yielded a non-significant result (intercept: 0.06; 95% CI: -3.89 to 4.02; t=0.032; p=0.977), indicating that publication bias is unlikely to have influenced the pooled outcome.

4

GRADE Certainty Assessment

The GRADE certainty assessment for the primary outcome (recurrence rate following surgical excision) is summarized in Supplement 2 (http://www.medicina.oral.com/carpeta/suppl2_27622). The overall certainty of evidence was rated as very Low.

## Discussion

The findings of this systematic review and meta-analysis offer critical insights into the recurrence rates and associated risk factors of localized juvenile spongiotic gingival hyperplasia following surgical excision. An overall pooled recurrence rate of approximately 7% suggests a relatively low risk of recurrence, although the observed variability across studies indicates that certain factors significantly influence outcomes (Figure 3).

The most salient finding from our analysis is the substantial impact of surgical technique on recurrence rates. Recurrence rates are approximately five-fold higher after incomplete or superficial excision techniques compared to complete excision with adequate margins. This suggests that the thoroughness of the surgical approach is a pivotal determinant of treatment success. The pooled recurrence rate of 7% aligns with the notion that while LJSGH is generally manageable through surgical means, the precision and extent of excision are crucial (1).

The temporal patterns of recurrence are noteworthy. Most recurrences, accounting for 68.4% of cases, occur within the first six months postoperatively, highlighting the necessity for close monitoring during this critical period. However, the occurrence of late recurrences, documented up to five years post-surgery, underscores the importance of long-term follow-up protocols (4). These findings suggest that while early postoperative care is crucial, extended surveillance is necessary to ensure comprehensive management of LJSGH.

The clinical implications of these findings are multifaceted. Primarily, complete excision with adequate margins should be considered the gold standard for treating LJSGH. Clinicians must be meticulous in ensuring that surgical margins are clear to minimize the risk of recurrence. Structured follow-up protocols extending beyond the initial six-month period are essential to detect and manage late recurrences effectively.

Alternative therapies, such as laser and cryotherapy, have shown promise in managing recurrent cases. These modalities may offer valuable adjuncts or salvage treatments, particularly in cases where traditional surgical approaches have failed or are contraindicated (27). Patient counseling should include thorough discussion of the potential for recurrence and the possible need for additional interventions, thereby setting realistic expectations and preparing patients for long-term management (20).

It is particularly relevant to note that the results of our pooled analysis are robust, especially considering the absence of small-study effects (Figure 3) and heterogeneity (Figure 4), despite the inherent limitations associated with sample size (28). This robustness in findings enhances the reliability of the observed recurrence rates and the factors influencing them. Monte Carlo meta-regression revealed no significant association between follow-up duration and recurrence rate. While this suggests that follow-up time may not strongly influence recurrence, the analysis is limited by the small number of studies, heterogeneous follow-up definitions, and the narrow distribution of recurrence rates. These inherent limitations reduce the power to detect subtle trends and warrant cautious interpretation.

Several limitations must be acknowledged despite the valuable insights provided by this meta-analysis. Most of the included studies were case reports or small case series, which inherently limit the statistical power and generalizability of the findings. The significant heterogeneity in surgical techniques, follow-up protocols, and outcome definitions across studies further complicates interpretation of the results (29). Selection bias is another critical limitation, as the published literature may overrepresent complicated or unusual cases, potentially skewing the perceived recurrence rates (30). Additionally, the variability in follow-up durations and frequencies across studies introduces inconsistencies that may affect the comparability of outcomes.

To address these limitations and build on the current findings, several future research directions are proposed. Prospective cohort studies with standardized surgical protocols and follow-up schedules are needed to provide more robust and generalizable data. Comparative effectiveness research evaluating different surgical techniques and adjuvant therapies will help identify the most effective treatment modalities. Long-term outcome studies with a minimum follow-up duration of two years are essential to fully understand the natural history of LJSGH and the factors influencing recurrence. Furthermore, biomarker studies aimed at identifying predictors of recurrence risk could provide valuable insights into the underlying mechanisms of LJSGH and potentially lead to the development of targeted therapies.

## Conclusions

Localized juvenile spongiotic gingival hyperplasia demonstrates a moderate recurrence risk following surgical excision, with significant variation based on surgical technique and follow-up duration. Complete excision appears crucial for preventing recurrence, while structured long-term follow-up is essential for optimal patient outcomes.

The GRADE assessment indicates very low certainty of evidence, primarily due to study design limitations, imprecision related to small sample sizes, and potential publication bias inherent in case report literature. Future prospective studies with standardized protocols are needed to develop evidence-based clinical guidelines and improve patient care.

## Figures and Tables

**Table 1:** Table Key characteristics of the studies included.

First Author	Year	Country	Study Design	Sample Size	Gender	Age	Follow-Up Period	Mean Follow-Up Period(months)	Recurrence	Recurrence Rate (%)	Notes/Comments	
Darling et al.	2007	Canada	Case Series	24 patients	12 males,12 females	5-28 years(mean 12 years)	Documentedin 14 patients(6 months-18 years)	~87 (estimated)	Yes, in 4 cases	16.7%	Follow-up only in 14/24 patients	

Solomon et al.	2013	USA	Case Series	3 patients	2 male,1 female	9-15 years	4-6 months	~5	Yes, in 2 cases	66.67%	Small sample size	

Chang et al.	2008	Taiwan, USA	Case Series	52 patients	36 females, 16 males	7-39 years(mean 11.8 years)	6 months to 5 years	~33	Yes, in 3 cases	5.8%	Wide follow-up range	

Innocentini et al.	2020	Brazil	Case Series	8 patients	4 males,4 females	6-24 years(mean 11.6 years)	4 to 15 months	28.5	Norecurrence	0%	NR	

Argyris et al.	2015	USA	Case Series	21 patients	14 males, 7 females	8-36 years(mean 13 years)	2 to 35 months	18.5	Yes, in 2 cases	9.5%	NR	

Goldberg et al.	2022	USA	Case Series	2 patients	2 females	11-13 years	6 to 36 months	21	Recurrence in 1 case	50%	Variable follow-up	
Nogueira et al.	2017	Brazil	Case Report	2 patients	2 females	9 and 11 years	5 and 6 months	5.5	Yes, in both	100%	Small sample size	

Kalogirou et al.	2017	Greece	Case Report	2 patients	2 males	12 years	12-17 months	15	Norecurrence	0%	Single case with medium follow-up	
Siamantas et al.	2018	Greece	Case Report	1 patient	1 female	19 years	15 months	15	Yes	100%	Single case	
MacNeill et al.	2011	USA	Case Report	1 patient	1 male	14 years	6 months	6	Norecurrence	0%	Single case	
Yang et al.	2024	China	Case Report	1 patient	1 female	10 years	6 months	6	Norecurrence	0%	Single case	
Delli et al.	2023	Netherlands	Case Report	1 patient	1 female	49 years	2 years	24	Norecurrence	0%	Single case	

Roberts et al.	2022	USA	Case Report	1 patient	1 male	56 years	4 months	4	Yes	100%	Single case, short follow-up	

NR stands for not reported.

## Data Availability

The datasets used and/or analyzed during the current study are available from the corresponding author.
